# Multi-phenotype genome-wide association studies of the Norfolk Island isolate implicate pleiotropic loci involved in chronic kidney disease

**DOI:** 10.1038/s41598-021-98935-4

**Published:** 2021-09-30

**Authors:** Ngan K. Tran, Rodney A. Lea, Samuel Holland, Quan Nguyen, Arti M. Raghubar, Heidi G. Sutherland, Miles C. Benton, Larisa M. Haupt, Nicholas B. Blackburn, Joanne E. Curran, John Blangero, Andrew J. Mallett, Lyn R. Griffiths

**Affiliations:** 1 School of Biomedical Sciences, Centre for Genomics and Personalised Health, Genomics Research Centre, Institute of Health and Biomedical Innovation, Queensland University of Technology (QUT), 60 Musk Ave., Kelvin Grove, QLD 4059 Australia; 2 Institute for Molecular Bioscience & Faculty of Medicine, The University of Queensland, Brisbane, QLD Australia; 3 Institute of Environmental Science and Research, Kenepuru, New Zealand; 4 School of Medicine, South Texas Diabetes and Obesity Institute, The University of Texas Rio Grande Valley, Brownsville, TX USA; 5 Department of Human Genetics, School of Medicine, The University of Texas Rio Grande Valley, Brownsville, TX USA; 6 Menzies Institute for Medical Research, University of Tasmania, Hobart, TAS Australia; 7 Department of Renal Medicine, Townsville University Hospital, Townsville, QLD Australia; 8 College of Medicine & Dentistry, James Cook University, Townsville, QLD Australia

**Keywords:** Computational biology and bioinformatics, Genetics, Diseases, Nephrology

## Abstract

Chronic kidney disease (CKD) is a persistent impairment of kidney function. Genome-wide association studies (GWAS) have revealed multiple genetic loci associated with CKD susceptibility but the complete genetic basis is not yet clear. Since CKD shares risk factors with cardiovascular diseases and diabetes, there may be pleiotropic loci at play but may go undetected when using single phenotype GWAS. Here, we used multi-phenotype GWAS in the Norfolk Island isolate (n = 380) to identify new loci associated with CKD. We performed a principal components analysis on different combinations of 29 quantitative traits to extract principal components (PCs) representative of multiple correlated phenotypes. GWAS of a PC derived from glomerular filtration rate, serum creatinine, and serum urea identified a suggestive peak (p_min_ = 1.67 × 10^–7^) that mapped to *KCNIP4*. Inclusion of other secondary CKD measurements with these three kidney function traits identified the *KCNIP4* locus with GWAS significance (p_min_ = 1.59 × 10^–9^). Finally, we identified a group of two SNPs with increased minor allele frequencies as potential functional variants. With the use of genetic isolate and the PCA-based multi-phenotype GWAS approach, we have revealed a potential pleotropic effect locus for CKD. Further studies are required to assess functional relevance of this locus.

## Introduction

Chronic kidney disease (CKD) is the gradual deterioration of kidney function or structure over at least 3 months^1^. CKD can result in end-stage kidney disease (ESKD) whereby kidney replacement therapy is required. CKD prevalence and burden is steadily rising, with an estimated 10–15% of the world’s population affected^2^. Increased serum levels of creatinine, cystatin C or urea are often used to indicate kidney dysfunction^3^. The best current marker of CKD is glomerular filtration rate (GFR), which can be directly measured using exogeneous markers or estimated (eGFR) based on concentrations of endogenous filtration markers such as serum creatinine or cystatin C^4^.

The pathophysiology underlying CKD is not yet fully understood, which has hindered the early detection and prevention of CKD as well as development of effective therapeutic treatments. Genome-wide association studies (GWAS) have identified a number of loci in relation to CKD, eGFR or complementary biomarkers, e.g. serum creatinine and blood urea^5–8^. A recent GWAS meta-analysis of eGFR (n = 765,348) identified 308 associated loci, which together explained 19.6% of eGFR heritability^5^. In addition, the GWASs to date have mostly focused on populations with European ancestry, hence the complete genetic architecture underlying CKD has not yet been established^2^.

Other chronic disorders such as diabetes, high blood pressure, and obesity exhibit co-morbidity with CKD^9,10^. Furthermore, many endophenotypes for CKD risk exhibit substantial intercorrelation. This suggests that a genetic commonality, perhaps acting via pleiotropic mechanisms, may play a role in CKD and related disorders. This is supported by a phenome-wide association study (PheWAS) that revealed association of eGFR index SNPs with 7 phenotypes out of 23 cardiovascular and diabetes-related traits^11^. Principal component analysis (PCA) of multiple correlated quantitative endophenotypes can capture important underlying structure in the phenotypic data, which when analysed as outcomes in GWASs may reveal loci that would remain undetected when traits are analysed individually. Avery et al.^12^ sucessfully identified three new loci associated with multiple-phenotype domains of metabolic syndrome by using the PCA-based GWAS approach. Applying the same method, Fatumo et al.^13^ identified new susceptibility genes for blood cell traits that were not identified in the standard univariate GWAS. Thus, investigating multiple CKD-related phenotypes via PCA might reveal new insights into the genetic basis of CKD.

The use of genetically isolated populations can empower genetic mapping studies of complex traits. Genetic isolates are often defined by founder effects resulting in reduced genetic diversity and increased frequency of variants that are rare in other populations^14^. Norfolk Island (NI) is a small and remote island located in the Pacific Ocean and is about 1400 km off the east coast of mainland Australia. Most of the modern-day Norfolk Island population are direct descendants of 11 European Mutineers of the HMS Bounty and 6 Polynesian women from the late eighteenth century. Thus, the NI population now exhibits founder effects, admixture and increased homozygosity. For almost 20 years, the NI isolate has been a valuable resource for genetic research^15–19^. Notably, a study of multiple cardiovascular disease (CVD) risk traits in the NI cohort group identified a potential pleiotropic effect locus on chromosome 1p22.2. This locus was only revealed from a GWAS of a multiple quantitative endophenotypes for CVD^19^.

In this study, we performed a GWAS of multiple phenotypes in 380 individuals of the NI isolate with the aim of identifying pleiotropic loci associated with CKD. The phenotypes included for CKD were primary traits, i.e. creatinine-based eGFR, serum creatinine level, and serum urea level, as well as 26 secondary phenotypes including anthropometric and biochemical measurements. We applied PCA on the 3 CKD-primary traits to identify components representing covariance among them and then performed GWAS on the principal components yielding statistically significant heritability. We also included 26 secondary CKD traits into the analysis based on correlation clusters and combined these with the primary traits to perform the same PCA and GWAS workflow. As a result, we were able to identify variants in the *KCNIP4* gene, which encodes for the Potassium Voltage-Gated (Kv) channel-interacting protein 4, as a potential pleiotropic locus of CKD. In addition, we also identified two SNPs with MAFs substantially enriched in NI isolate to be the potential functional variants.

## Results

### Epidemiology of CKD risk in NI

**Figure 1:**
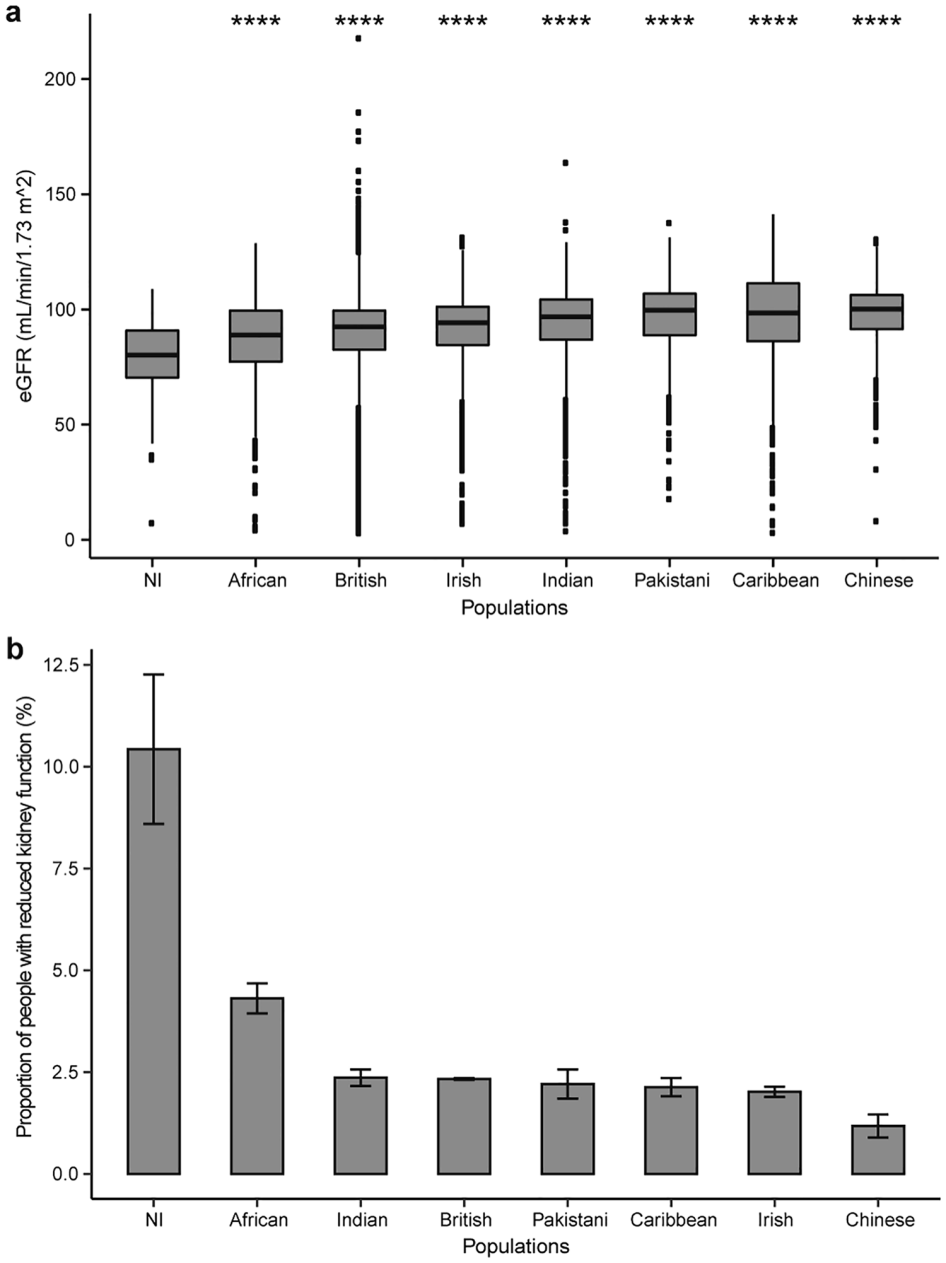
Comparison of eGFR in NI isolate to those of other populations. (**a**) Distributions of eGFR in NI isolate and other populations. There were statistically significant differences between mean of eGFR in NI compared to those of other population (adjusted P-values < 2e−13). (**b**) Proportion of people with reduced kidney function (eGFR < 60 mL/min/1.73 m^2^) in each population. Data are represented as the percentage ± SE.

To compare eGFR levels of the NI isolate to those of other populations, we obtained CKD-related phenotypic data from the UK Biobank^20^ (UKBB) (Fig. 1). The number of individuals and age range in each ethnicity can be found in Supplementary Table S1. To ensure the NI data was comparable to the UKBB data in terms of age range we included only NI individuals aged between 40 and 70 years, comprising 278 samples. Out of all the populations, the NI isolate exhibited the lowest eGFR profile (Fig. 1a). Further examination of eGFR that was less than 60 mL/min/1.73 m^2^, a level indicative of reduced kidney function, showed the NI cohort as the population with the highest proportion in this category (Fig. 1b). Collectively, eGFR levels in the NI isolate indicated a very high degree of potential kidney disease prevalence when compared to other world populations. Importantly, the actual prevalence of CKD in NI may be even higher as individuals with end-stage renal disease leave the island for mainland Australia to receive treatments such as renal transplant or dialysis.

The high rate of reduced kidney function observed in the NI isolate may also reflect the influence of Polynesian ancestry in the gene pool. We note that there is not comparable eGFR data available for Polynesian populations, however, many studies have shown that the prevalence of ESKD is much higher in Polynesian populations in comparison to Europeans^21^.

### Single phenotype analysis

Serum creatinine, eGFR, and serum urea were 3 CKD-primary traits available in the NI isolate phenotypic collection. According to the literature^22^, heritability estimates of eGFR, serum urea, and serum creatinine were 44%, 31%, and 37% respectively. In our study, these traits showed lower heritability estimates, ranging from 0.27 to 0.3 (P < 0.05) (Supplementary Table S2). GWAS of these individual traits did not identify loci passing the genome-wide significance P-value threshold (results not shown).

### Principal components of the 3 CKD-primary traits

A PCA of serum creatinine, eGFR, and serum urea (CGU) generated 3 principal components (PC): CGU-PC1, CGU-PC2, and CGU-PC3 (Table 1). CGU-PC1 explained approximately two thirds of the total variance. For this component, serum creatinine and serum urea were found to be highly correlated to each other, but both were negatively correlated to eGFR. In relation to kidney diseases, low eGFR and high serum urea and creatinine levels are indicative of impaired kidney function. CGU-PC2 was also significantly correlated to all the 3 traits, with a particularly high contribution from serum urea levels; samples with high CGU-PC2 scores exhibited high serum urea, but relatively normal levels of creatinine and eGFR. As for the last component, CGU-PC3 accounted for the positive correlation between eGFR and creatinine, without inclusion of urea. The individual component map under CGU-PC1 and CGU-PC2 is illustrated in Supplementary Fig. S1.

**Table 1:** Statistics of principal components extracted from eGFR, serum creatinine, and serum urea.

Principal component	% Variance	Loadings			Heritability	
		eGFR	Serum creatinine	Serum urea	${h}^{2}$	P-value
CGU-PC1	66	− 0.600	0.607	0.522	0.33	7.75 × 10^–4^
CGU-PC2	21.1	0.406	− 0.331	0.852	0.26	6.66 × 10^–3^
CGU-PC3	12.9	0.690	0.723	− 0.048	0.12	1.35 × 10^–1^

### Heritability and GWAS analysis of CKD components

Heritability estimation showed that only CGU-PC1 and CGU-PC2 had statistically significant heritability of 0.26 and 0.33, respectively (P < 0.05).

**Figure 2:**
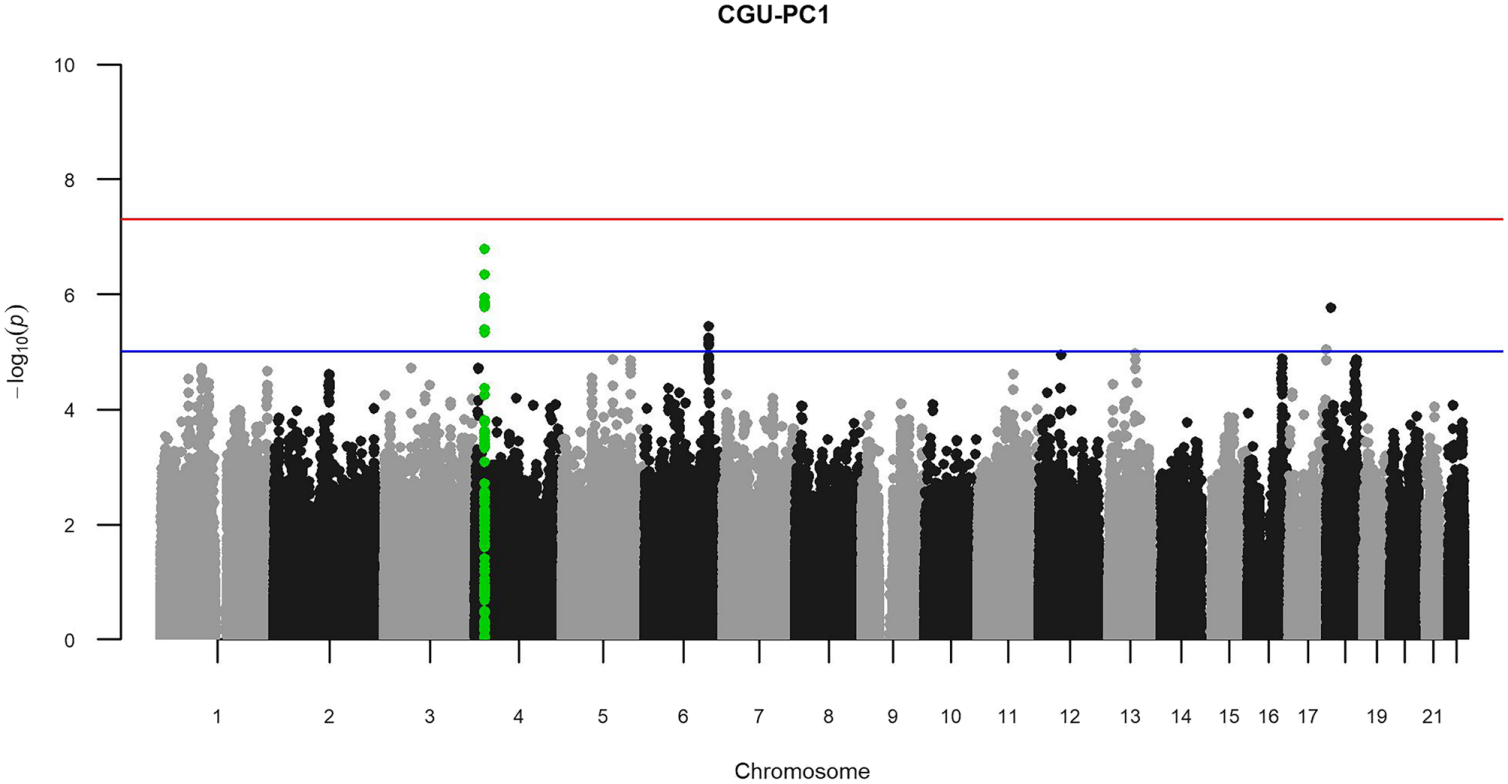
Manhattan plot for CGU-PC1 extracted from the 3 CKD primary traits. The red line indicates genome-wide significance threshold of 5 × 10^−8^, while the blue line indicates the suggestive threshold of 1 × 10^−5^. CGU—serum creatinine, eGFR, and serum urea.

GWASs of CGU-PC1 and CGU-PC2 did not identify any genome-wide significant loci. However, there was a cluster of SNPs mapped to the *KCNIP4* gene on chromosome 4 forming a clear GWAS peak for CGU-PC1 (Fig. 2, lead SNP rs12640604-A, MAF = 0.355, beta =  − 0.344, se = 0.066, P = 1.67 × 10^–7^). Given the high capacity of CGU-PC1 in explaining kidney dysfunction and the negative effect size of the lead SNP, the identified *KCNIP4* locus could be relevant in decreased susceptibility of CKD.

### Combinations of the 3 CKD traits and other measurements

Following analysis of the three primary CKD traits, we expanded the analysis to include other CKD-secondary phenotypes. Along with eGFR, serum creatinine, and serum urea, there were 26 other continuous measurements that were assessed (Supplementary Table S3).

Whether negatively or positively, correlated traits tend to gather into the same PCs. Therefore, we first clustered all the 29 continuous traits into groups based on significant bivariate correlations (P ≤ 0.05, Supplementary Table S4). Phenotypic traits indicative for specific metabolic functions were highly correlated to each other. For example, the three liver enzyme measurements: alkaline phosphatase, alanine aminotransferase, and gamma-glutamyl transferase all clustered into the same group. We also included the three CKD-primary phenotypes in the correlation analysis to see their relationships to each other and to the remaining phenotypes. The three traits clustered well into a single group as they are the biomarkers of renal function. The clusters were then combined with the three CKD-primary traits for PCA as well as further downstream analyses.

**Figure 3:**
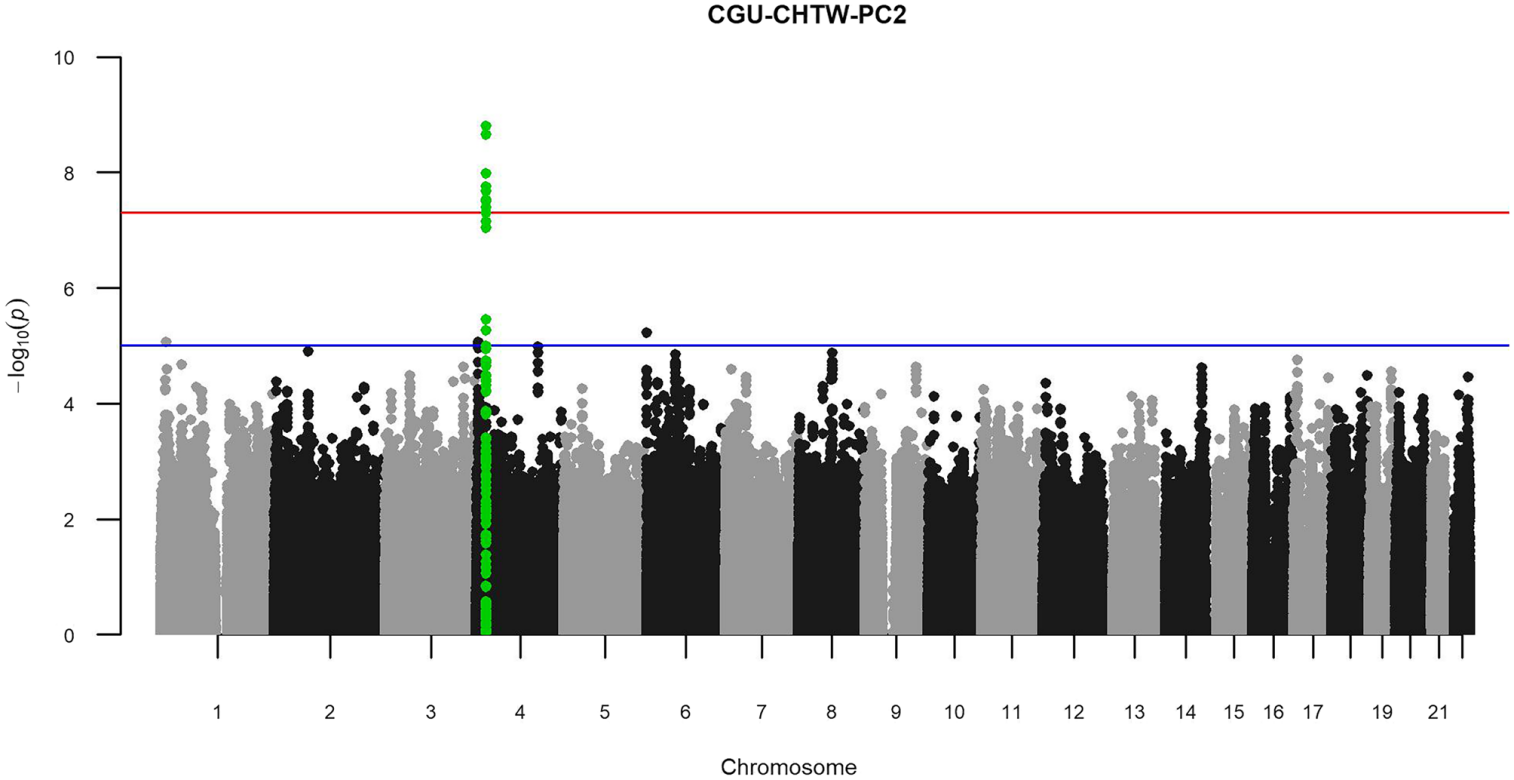
Manhattan plot for CGU-CHTW-PC2 showing *KCNIP4* peak with higher significance than in the GWAS of CGU-PC1. The red line indicates the genome-wide significance threshold of 5 × 10^−8^, while the blue line indicates the suggestive threshold of 1 × 10^–5^. *CGU* serum creatinine, eGFR, and serum urea, *CHTW* cholesterol HDL-C ratio, HDL-cholesterol, triglyceride, and waist hip ratio.

In total, there were ten combinations included for further analyses (Supplementary Table S4). From a total of 58 generated combination PCs, 30 showed statistically significant heritability estimates and significant association peaks were identified in three PCs (Table 2). Notably, the *KCNIP4* gene was identified in two of those. Specifically, a *KCNIP4* association was found in PCs extracted from the three CKD-primary traits combined with other secondary traits as follows: total cholesterol (TC)/HDL-C ratio, HDL, triglyceride, and waist hip ratio (i.e. CGU-CHTW-PC2); body fat and height (i.e. CGU-BH-PC1). Of note, the association of *KCNIP4* in the CGU-CHTW-PC2 combination was identified with the the lowest P-value of p_min_ = 1.59 × 10^–9^ (Fig. 3).

**Table 2:** Combination PCs with genome-wide significant association peaks.

Principal component	Chr.	SNP	BP	A1	A2	Freq.	Beta	P-value	Gene
CGU-AT-PC3	2	rs17863787	233,702,448	G	T	0.314	0.603	1.18 × 10^–13^	*UGT1A8*
CGU-BH-PC1	4	rs12640604	20,999,244	A	G	0.355	− 0.347	3.37 × 10^–8^	*KCNIP4*
CGU-CHTW-PC2	4	rs12640604	20,999,244	A	G	0.355	− 0.448	1.59 × 10^–9^	*KCNIP4*

Further analysis of the index SNP (rs12640604) with all the 29 phenotypic traits showed this locus to be highly associated with eGFR, creatinine, and urea (Supplementary Fig. S2). The inclusion of more correlated traits in PCs might increase the statistical power to identify susceptible loci in GWAS. We examined the trait correlation with component variables in each PC (Supplementary Table S5). CGU-PC1, CGU-CHTW-PC2, and CGU-BH-PC1 all had very similar contributions to the three CKD-primary traits, i.e. the PCs were all positively correlated to serum creatinine and eGFR as well as negatively correlated to serum urea. We looked at other PCs with similar profiles. Interestingly, we found that the sum of all the correlation coefficients was highly correlated to the corresponding GWAS P-value (Supplementary Fig. S3). Therefore, the observed increase in the statistical power to identify loci can be attributed to the decrease in total correlation coefficients in the PCs. Conversely, some trait combinations, e.g. BMI, hip circumference, waist circumference, and weight (CGU-BHWW-PC1 in Supplementary Table S5), when added to the three CKD-primary traits increased the sum of all the correlation coefficients between the variables and the PCs, and hence resulted in a high GWAS P-value. It should also be noted that the increased power achieved was not due to increased heritability of the combined PCs (Pearson correlation =  − 0.29).

The inclusion of both serum creatinine and eGFR in all the PCAs was unnecessary as eGFR was estimated from creatinine, along with age and sex information. We examined whether removing serum creatinine out of the PCAs would alter the GWAS results. Consequently, the *KCNIP4* peak was still detected and rs12640604 continued to be the most significant SNP in all of these additional GWASs (Supplementary Table S6).

Along with *KCNIP4*, we also identified suggestive genome-wide significance at the *UGT1A8* locus in the GWAS of CGU-AT-PC3 (derived from the three CKD-primary traits, albumin, and total bilirubin). *UGT1A8* is an established bilirubin-associated gene.

### Replication analysis

We utilized the UKBB data to test for replication of the association between the index SNP for *KCNIP4* gene (rs12640604) and CGU-PC1 extracted from the three CKD-primary traits. The UKBB is comprised of multiple different ethnic (and ancestral) subgroups and these were analyzed separately (Supplementary Table S7). The MAF for rs12640604 in each of the UKBB ethnicities are shown in Table 3 and were not substantively different to NI. In each subgroup, CGU-PC1 was newly constructed using the same formula developed for the NI cohort. Similar to the CGU-PC1 in the NI isolate, all the components in the UKBB populations were positively correlated to serum creatinine and urea, while negatively correlated to eGFR (Table 3). In the Caribbean and Indian subgroups, rs12640604-A and CGU-PC1 showed some evidence of association (P-values < 0.05). However, the effect size in the NI isolate (beta =  − 0.313 and P-value = 1.38 × 10^–6^) was several times higher than those in the Caribbean and Indian. Finally, only the Indian subgroup effect size was in the same direction as that in the NI isolate. Of note, the index SNP showed some evidence for association to the individual CKD traits in several subgroups (P < 0.05) although the modest level of statistical significance would not implicate this SNP in large GWASs.

**Table 3:** Association analyses of rs12640604-A and in UKBB data and CGU-PC1 in each UKBB subgroup.

Principal components	Loadings			Association with rs12640604-A		
	eGFR	Creatinine	Urea	Allele frequency	BETA	P
African-CGU-PC1	− 0.577	0.598	0.556	0.614	–	–
British-CGU-PC1	− 0.605	0.6	0.524	0.379	–	–
Caribbean-CGU-PC1	− 0.581	0.6	0.55	0.578	0.064	0.047
Chinese-CGU-PC1	− 0.599	0.598	0.533	0.388	–	–
Indian-CGU-PC1	− 0.591	0.59	0.55	0.274	− 0.07	0.022
Irish-CGU-PC1	− 0.604	0.599	0.526	0.386	–	–
Pakistani-CGU-PC1	− 0.602	0.608	0.518	0.258	–	–

(–) Association results with P-values > 0.05.

### Potential functional variants in KCNIP4

In the NI cohort we identified 8940 SNPs spanning *KCNIP4* of which*,* there were 47 SNPs that exhibited some LD (R^2^ > 0.2) with the index SNP (rs12640604). These SNPs showed MAFs ranging from 0.146 to 0.491. Comparison with the MAFs taken from the UKBB, in which 46 SNPs were available, showed that most of the SNPs had comparable MAFs to NI cohort (the ratios of NI MAFs to the UKBB MAFs ranged from 0.6 to 2.3). In contrast, two SNPs (rs148583816 and rs143182955), which are a few base pairs apart from one another, have MAFs approximately tenfold higher than their MAFs in NI isolate (Table 4). These SNPs were part of an LD region spanning approximately 37.8 Mb in the NI cohort. However, in the UKBB, rs148583816 and rs143182955 were not in LD (LD < 0.2) with the index SNP.

**Table 4:** Statistics of the two potential functional SNPs.

ID	Chr.	Position	Ref	Alt	Compared to the index SNP rs12640604			
					Distance	R^2^	D'	
rs148583816	4	20,999,427	G	T	183	0.3363	0.6434	
rs143182955	4	20,999,431	G	T	187	0.2492	0.5334	

ID	MAF in the NI isolate	MAF in the UK biobank						
		British	Chinese	Irish	Indian	Caribbean	African	Pakistani
rs148583816	0.458	0.044	0.048	0.043	0.043	0.092	0.093	0.04
rs143182955	0.443	0.044	0.039	0.04	0.026	0.015	0.012	0.026

Examining these 2 SNPs in the UCSC Genome Browser^23^, we observed that they are located on a T-enriched region and coincided with a histone modification H3K4me3. H3K4me3, or tri-methylation of lysine 4 on histone H3, is a chromatin modification at the transcription start site and its level is positively associated with transcription^24^. For *KCNIP4*, several transcripts have been identified, some of which are expressed in the human kidney^25^. Interestingly, the associated SNPs are located on the promoter region of isoform *KCNIP4-IeΔII* which is one of the transcripts expressed in the kidney along with *KCNIP4-IbΔII* and *KCNIP4-IcΔII*. Taken together, these SNPs may play a regulatory role in *KCNIP4* gene expression due to founder effect in NI.

### Spatial gene expression of KCNIP4

**Figure 4:**
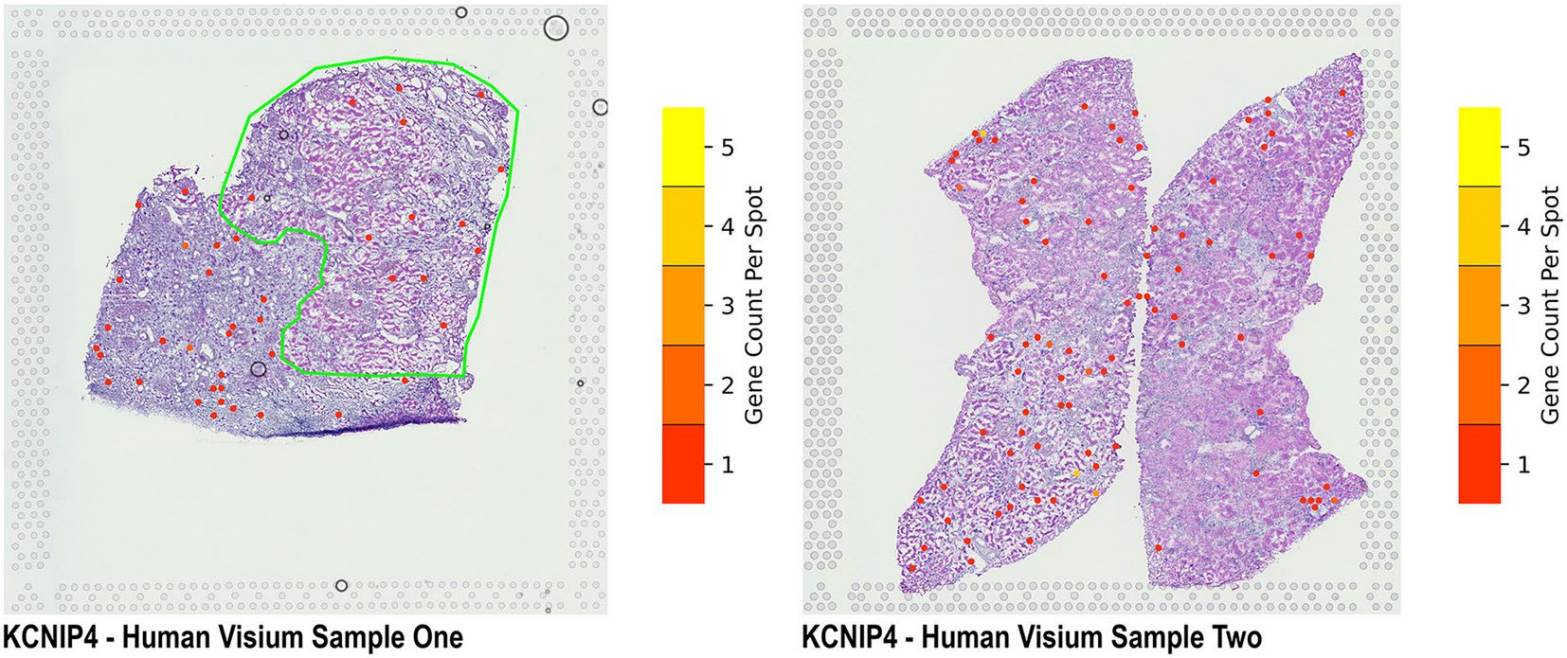
Spatial gene expression of *KCNIP4* in human cortical kidney tissue sections. We localized *KCNIP4* gene expression within the tubules of both sample one, composed of non-scarred (green) and scarred cortical regions and sample two, composed of non-scarred cortical region.

Using Visium Spatial Gene Expression (10 × Genomics), we localized the expression of *KCNIP4* gene to the tubules, in both non-scarred and scarred human cortical kidney tissue sections (Fig. 4). Furthermore, we found no expression of the *KCNIP4* gene in the glomeruli or vasculature of the non-scarred and scarred human kidney tissue sections.

## Discussion

For the past decade, multiple genetic loci associated with kidney function have been identified via GWAS. However, the genetic basis of CKD is still not completely understood and requires a range of approaches to tackle the problem. In this study, we aimed to identify novel susceptible loci underlying kidney function using a multi-phenotype approach in combination with the use of the genetically isolated cohort of Norfolk Island (NI). As a result, the *KCNIP4* locus was identified in principal component traits derived from three CKD-primary traits as well as with various secondary trait combinations. The three derived composite phenotypes, i.e. CGU-PC1, CGU-BH-PC1, and CGH-CHTW-PC2, have encapsulated information that were not entirely expressed in the individual traits, hence, we were able to identify the *KCNIP4* locus in these components but not in any single-phenotype.

The gene *KCNIP4* which encodes for the Potassium Voltage-Gated (Kv) channel-interacting protein 4 has several transcripts with varied cellular expressions, and potentially different protein functions. In kidney, the three active transcripts are *KCNIP4-IbΔII*, *KCNIP4-IcΔII*, and *KCNIP4-IeΔII*^25^. Previously, the disruption of *KCNIP4* has been observed in patients with renal-cell carcinoma. This is possibly due to alteration of the normal transcriptional regulation since the breakpoint interval coincides with the promoter of transcript *KCNIP4-IcΔII*^26^. In our study, the associated SNPs were located in the region corresponding to the promoter of the transcript *KCNIP4-IeΔII*, hence the associated variants may have a regulatory role in gene expression. In a recent publication, Gerhardt et al.^27^ found that *KCNIP4* was markedly up-regulated at the late injury stage in proximal tubule cells in an ischemia–reperfusion injury model for studying acute kidney injury (AKI). Kidney proximal tubule is particularly vulnerable to injury and maladaptive repair of injured tubules after AKI can lead to CKD^28,29^. Therefore, the up-regulation of *KCNIP4* can be one of the factors involved in the consistent impairment of the proximal tubule in kidney injury. In support of this we were also able to identify spatial gene expression of *KCNIP4* in two human cortical kidney tissue sections. In these samples, *KCNIP4* expression was localized to the tubules, with no expression in the glomeruli or vasculature of the cortical kidney tissue.

To pinpoint the potential regulatory SNPs, we analyzed all the SNPs in LD with the index SNP and found two nearby SNPs with MAFs much greater in NI isolate compared to other world populations. Consistent with the hypothesis that the associated SNPs involved in transcriptional regulation, this group was in a region where the histone modification H3K4me3 occurred. The founder effect of NI isolate, along with the high inbreeding rate, has mostly likely increased the frequencies of these alleles, which in turn has increased the power to detect the association relative to other major ancestral populations.

One of the limitations of the study was the lack of urine biomarkers for measuring albuminuria, which are especially important to detect CKD when eGFR is in normal range^3^. However, because the initial aim of the Norfolk Island Health Study (NIHS) was to study cardiovascular diseases, only serum biomarkers were collected. Also, the inclusion of both eGFR and serum creatinine in PCA was not entirely justified because these were highly correlated variables i.e. eGFR was calculated based on serum creatinine, age, and sex. However, the exclusion of serum creatinine did not alter the current findings.

In conclusion, the use of the NI genetic isolate in combination with the PCA-based multi-phenotype approach revealed the *KCNIP4* locus to be associated with CKD. Two associated variants which minor allele frequencies that were about tenfold higher in the NI isolate than in other world populations potentially play a regulatory role in the *KCNIP4* gene expression. Further studies are needed to assess the biological functions of the identified variants in relation to CKD.

## Materials and methods

### Norfolk Island Health Study

The Norfolk Island Health Study (NIHS) is a well-established study aimed at identifying genetic and environmental risk factors for CVD and related diseases^16,17,30^.

In this study, we included 380 individuals with available genomic and phenotypic data. These individuals consisted of 196 females (mean age: 49.8 ± 16.5) and 184 males (mean age: 47.1 ± 15.3) all of whom were members of the core-pedigree and at least 18 years of age at the time of collection. Serum samples were drawn from each individual to measure serum biomarkers as well as to collect blood-based DNA.

Ethical approval was granted prior to the commencement of the study by the Griffith University Human Research Ethics Committee (approval no: 1300000485). Ethics approval and management of the NIHS has since been transferred to Queensland University of Technology (approval no: 1600000464). All individuals gave written informed consent prior to the study and all methods were carried out in accordance with relevant guidelines and regulations.

### Principal component analysis of CKD endophenotype data

In total there were 29 quantitative phenotypic traits (Supplementary Table S3) along with age and sex information of 380 NIHS individuals included in this study. eGFR was calculated using the CKD-EPI Creatinine equation^31^. Missing data were imputed using the MissMDA 1.3 package^32^. The package can perform multiple imputation using principal component analysis (PCA), meaning the imputed values will not affect PCA results.

After the imputation of missing data, the PCA method was applied to transform multi-dimensional data into fewer components. PCA is one of the optimal approaches in multiphenotype analysis, especially in the case of association studies^33^. Principal component (PC) analyses were performed with the package FactoMineR 1.42^34^, which integrated multiple PCA exploratory methods and illustrations.

### Genome-wide SNP genotyping

NI genomic data were generated from two platforms: Illumina HiSeq X10 sequencing (n = 108) and Illumina 610-Quad array (n = 506). Whole genome sequencing (WGS) directly revealed nearly 20 million SNPs, while the SNP-array, which was imputed using the 1000 genomes project^35^ as a reference, identified over 26 million SNPs. In total, genomic data was available for 520 individuals.

To merge the SNP-array and WGS data, the common SNPs across both data sets were identified, and data associated with the common SNPs extracted. The merge procedure was performed using the default parameters of the ‘bmerge’ function in PLINK 1.9^36^. For overlapping individuals, WGS data was given preference such that SNP-array data was removed.

Quality control filters were applied to the unified genomic data set to ensure high quality SNP data for subsequent analysis. A P-value of 1.84 × 10^–7^ was applied as the HWE threshold—a specific value calculated to account for widespread linkage disequilibrium in the NI cohort. A 5% missing genotyping filter was also applied on individuals and variants to ensure high quality data for subsequent analysis. Finally, variants with a minor allele frequency less than 0.05 and samples without phenotype data were removed. A total of 380 individuals and 4,753,086 SNPs remained after filtering.

### Heritability estimation

We utilized the SOLAR 8.5.1 program^37^ to estimate heritability for each individual trait and phenotypic PCs. The extended pedigree information of the NI isolate^38^ was integrated to account for the high degree of relatedness among the samples. All traits and PCs with a high excess kurtosis were inverse-normal transformed prior to heritability estimation.

### Genome-wide association analysis

Genome-wide association analysis (GWAS) was only performed for traits that yielded heritability estimates that were statistically significant at a nominal level (P < 0.05). To account for the family relatedness in association testing, we applied the mixed linear model, where a genetic relationship matrix (GRM) containing all the genotype correlations between all pairs of individuals was fitted as a random effect with age and sex fitted as additional fixed effects when computing the associations between phenotype and genetic markers. However, tested SNPs were excluded from calculating the GRM as implemented in the GCTA-LOCO approach^39^ to avoid loss of power when double-fitting of the candidate variants in the model.

We examined the inclusion of principal components of all genotyped SNPs’ GRM as a common method to address confounding due to population structure. PCA was performed with PLINK 1.9 and the first numbers of PCs were included as covariates along with age and sex in the GWAS. We found that as more PCs were included, the deflation in the statistics tests increased (Supplementary Fig. S5). However, further testing showed that the association signal from the top (index) SNP was not appreciable changed by the inclusion of PCs 1 and 2 (Supplementary Table S4). Given the modest sample size used in this study and the risk of overburdening the GWAS model with covariates we chose to omit genomic PCs from the primary analysis.

We also applied a filter to remove loci due to spurious associations. Since there is extended LD present in the NI cohort, we reasoned that associated SNPs would mostly be in LD with other associated SNPs located nearby. Thus, significantly associated loci were deemed as those that passed the genome-wide significance P-value threshold of 5 × 10^–8^, as well as having 2 or more variants within a 50 kb distance and with P-value ≤ 1 × 10^–5^. LD tests were performed with PLINK 1.9.

### Fine mapping

We used the WGS data of 108 NI samples to explore allele frequencies of variants that are in LD with the GWAS most associated SNP rs12640604 at the *KCNIP4* locus. Minor allele frequencies (MAFs) of these SNPs were then compared to those in the gnomAD database (v3.1.1)^40^ and the UKBB data. Genes and SNPs were visualized on the UCSC Genome Browser.

### Visium spatial gene expression

Existing Visium spatial gene expression data of two human cortical kidney tissue section^41^ were used to identify the spatial gene expression of *KCNIP4*. These data were visualized using the gene_plot function of stLearn^42^ and compared to existing data from the Human Protein Atlas.

### Replication analysis

We performed association analysis between the index SNP mapped to the *KCNIP4* gene and CGU-PC1 extracted from the 3 CKD-primary traits in populations included in the UKBB^20^ data (Supplementary Table S5). Serum creatinine and serum urea were available while creatinine-based eGFR was calculated using the CKD-EPI formula^31^. CGU-PC1 were newly constructed for each sub-group. Genotypic association analysis for each population was performed with simple linear models using the allele counts of rs12640604-A as independent variable and CGU-PC1 as the outcomes.

## Supplementary Information

Supplementary Information.

## Data Availability

The genotypic and phenotypic data of the Norfolk Island isolate supporting the current study are not publicly available due to ethics constraints but are available from the corresponding author on reasonable request.
