# Supplementary material for: Multi-phenotype genome-wide association studies of the Norfolk Island isolate implicate pleiotropic loci involved in chronic kidney disease
Source: Sci Rep. 2021 Sep 30;11:19425. doi: 10.1038/s41598-021-98935-4 (PMC8484585; doi:10.1038/s41598-021-98935-4)
Supplement: Supplementary file 1 — Supplementary Information. [file 41598_2021_98935_MOESM1_ESM.pdf]

Supplementary Information

Multi-phenotype genome-wide association studies of the Norfolk Island isolate implicate pleiotropic loci involved in chronic kidney disease

Submitted to Scientific Report

Authors

Ngan K. Tran,[1] Rodney A. Lea,[1] Samuel Holland,[2] Quan Nguyen,[2] Arti M. Raghubar,[2] Heidi G. Sutherland,[1] Miles C. Benton,[3] Larisa M. Haupt,[1] Nicholas Blackburn[4,5,6], Joanne E. Curran[4,5], John Blangero[4,5], Andrew J. Mallett,[2,7,8] and Lyn R. Griffiths[1,*]

Affiliations

[1]Queensland University of Technology (QUT), Centre for Genomics and Personalised Health, Genomics Research Centre, School of Biomedical Sciences, Institute of Health and Biomedical Innovation, 60 Musk Ave., Kelvin Grove, Queensland 4059, Australia

[2] Institute for Molecular Bioscience & Faculty of Medicine, The University of Queensland, Brisbane, QLD, Australia

[3] Institute of Environmental Science and Research, Kenepuru, New Zealand

[4]South Texas Diabetes and Obesity Institute, School of Medicine, The University of Texas Rio Grande Valley, Brownsville, Texas, USA

[5]Department of Human Genetics, School of Medicine, The University of Texas Rio Grande Valley, Brownsville, Texas, USA

[6]Menzies Institute for Medical Research, University of Tasmania, Hobart, TAS, Australia

[7] Department of Renal Medicine, Townsville University Hospital, Townsville, QLD, Australia

[8] College of Medicine & Dentistry, James Cook University, Townsville, QLD, Australia

Correspondence

***** lyn.griffiths@qut.edu.au

Table S1. Summarized information of 7 populations in the UK Biobank data used in the Epidemiology of CKD risk analysis.

Ethnic groups	n	Minimum age	Maximum age
British	414344	38	73
Irish	12290	40	70
Indian	5497	40	72
Caribbean	4127	39	70
African	3040	40	70
Pakistani	1674	40	70
Chinese	1443	40	70

Table S2. Heritability estimates for CKD-primary traits in the study.

Trait	Heritability	P-value
Serum creatinine	0.30	5.76×10^{-3}
eGFR	0.27	8.77×10^{-3}
Serum urea	0.30	8.62×10^{-4}

Table S3. Descriptive statistics for 29 traits of Norfolk Island isolate.

No.	Trait	Description	Unit	Minimum	Median	Mean	Maximum
1	ALBU	albumin	g/L	33	42	41.83	52
2	ALKPHS	alkaline phosphatase	U/L	5	63	65.84	169
3	ALT	Alanine Aminotransferase	U/L	7	17	19.99	97
4	BF	body fat	(%)	9	31	30.5	53.5
5	BICARB	bicarbonate	mmol/L	21	29	28.78	37
6	BMI	body mass index	.	15.02	26.39	26.9	54.47
7	CHLOR	chloride (Cl)	mmol/L	92	102	102.2	112
8	CHOL	total cholesterol	mmol/L	2.9	5.6	5.693	9.9
9	CHOLHDL	cholesterol HDL-C ratio	.	1.72	4.14	4.268	7.98
10	CREAT	serum creatinine	µmol/L	50	80	82.57	188
11	DBP	mean diastolic blood pressure	mm Hg	46	77	78.54	120
12	eGFR	estimated glomerular filtration rate	mL/min	34.84	86.07	85.29	134.73
13	GGT	Gamma-glutamyl Transferase	U/L	7	20	29.84	237
14	GLOB	globulins	g/L	20	30	30.45	42
15	GLU	random glucose	mmol/L	3.6	5.4	5.524	9.7
16	HDL	HDL-cholesterol	mmol/L	0.67	1.355	1.408	2.6
17	HEIGHT	height	cm	1.47	1.7	1.704	1.94
18	HIP	hip circumference	cm	76	102	102.8	146
19	LDL	LDL-cholesterol	mmol/L	0.71	3	3.046	7.3
20	POTA	potassium (K)	mmol/L	1.2	4.2	4.255	9.9
21	SBP	mean systolic blood pressure	mm Hg	75	128	130.9	210
22	SODIUM	sodium (Na)	mmol/L	132	140	140.5	162
23	TBILI	bilirubin (total)	µmol/L	1.8	7	8.507	38
24	TPROT	total protein (obtained by adding ALB and GLOB)	g/L	54	72	72.29	88
25	TRIG	triglyceride	mmol/L	0.1	1.6	1.874	6.5
26	UREA	urea	mmol/L	1.8	5.5	5.616	10.9
27	WC	waist circumference	cm	59	88	88.99	143
28	WEIGHT	weight	kg	44.8	77.5	78.25	166.8
29	WHR	waist hip ratio	.	0.66	0.87	0.8631	1.08

Table S4. Combination principal components with significant heritability ($P < 0.05$).

Primary traits	Secondary traits	Principal components	Heritability	P-value
eGFR, Creatinine, and Urea	Globulins, Total protein	CGU-GT-PC1	0.36	4.49E-04
		CGU-GT-PC2	0.25	6.15E-03
		CGU-GT-PC3	0.29	2.85E-03
	Alkaline phosphatase, Alanine aminotransferase, Gamma-glutamyl Transferase	CGU-AAG-PC1	0.28	7.18E-03
		CGU-AAG-PC2	0.27	1.12E-02
		CGU-AAG-PC3	0.39	9.61E-05
		CGU-AAG-PC4	0.53	5.09E-05
	Albumin, Total bilirubin	CGU-AT-PC1	0.34	5.77E-04
		CGU-AT-PC3	0.43	8.21E-05
		CGU-AT-PC4	0.28	1.72E-03
	Glucose, Potassium	CGU-GP-PC1	0.32	1.08E-03
	Chloride, Sodium	CGU-CS-PC1	0.29	2.00E-03
	Body fat, Height	CGU-BH-PC1	0.3	1.81E-03
		CGU-BH-PC2	0.53	3.40E-06
		CGU-BH-PC3	0.3	2.13E-03
		CGU-BH-PC4	0.51	2.00E-07
	BMI, Hip circumference, Waist circumference, Weight	CGU-BHWW-PC1	0.44	6.30E-06
		CGU-BHWW-PC2	0.32	5.02E-04
		CGU-BHWW-PC3	0.23	1.34E-02
		CGU-BHWW-PC7	0.71	4.16E-09
	Cholesterol HDL-C ratio, HDL-cholesterol, Triglyceride, Waist hip ratio	CGU-CHTW-PC2	0.37	2.20E-04
		CGU-CHTW-PC4	0.29	2.47E-03
		CGU-CHTW-PC5	0.32	2.28E-03
		CGU-CHTW-PC7	0.31	3.09E-03
	Total cholesterol, LDL-cholesterol	CGU-CL-PC1	0.37	4.95E-04
		CGU-CL-PC2	0.29	1.45E-03
		CGU-CL-PC3	0.28	4.16E-03
	Diastolic blood pressure, Systolic blood pressure	CGU-DS-PC1	0.23	9.80E-03
		CGU-DS-PC2	0.32	1.15E-03
		CGU-DS-PC3	0.25	7.52E-03

Table S5. Association results of the *KCNIP4* locus - lead SNP rs12640604 - in 10 combination PCs and CGU-PC1. The trait correlation coefficients of component variables were included for each PC. There was high correlation between GWAS p-values and the sums of coefficients (R=0.883).

PC	GWAS - rs12640604			Trait correlation coefficients							
	Beta	SE	P-value	CREAT	UREA	eGFR	Trait 3	Trait 4	Trait 5	Trait 6	Total
CGU-CHTW-PC2	-0.448	0.074	1.59E-09*	0.595	0.524	-0.712	0.548 (HDL)	-0.336 (TRIG)	-0.360 (CHOLHDL)	NA (WHR)	0.258
CGU-BH-PC1	-0.347	0.063	3.37E-08*	0.909	0.719	-0.722	0.290 (HEIGHT)	-0.252 (BF)			0.944
CGU-GT-PC1	-0.368	0.070	1.20E-07	0.801	0.655	-0.750	0.456 (TPROT)	0.450 (GLOB)			1.612
CGU-PC1	-0.344	0.066	1.67E-07	0.854	0.734	-0.844					0.744
CGU-AT-PC1	-0.344	0.066	1.87E-07	0.856	0.731	-0.843	NA (ALB)	NA (TBILI)			0.745
CGU-GP-PC1	-0.336	0.065	2.57E-07	0.847	0.718	-0.845	0.240 (POTA)	0.187 (GLU)			1.147
CGU-AAG-PC1	-0.321	0.063	3.23E-07	0.844	0.684	-0.769	0.408 (ALKPHS)	0.319 (GGT)	0.233 (ALT)		1.719
CGU-CS-PC1	-0.321	0.066	1.32E-06	0.842	0.711	-0.821	0.295 (SODIUM)	0.239 (CHLOR)			1.265
CGU-DS-PC1	-0.217	0.061	3.36E-04	0.703	0.659	-0.740	0.770 (SBP)	0.674 (DBP)			2.067
CGU-CL-PC1	-0.196	0.065	2.70E-03	0.684	0.546	-0.757	0.781 (CHOL)	0.733 (LDL)			1.987
CGU-BHWW-PC1	-0.176	0.067	8.72E-03	0.507	0.411	-0.381	0.870 (HIP)	0.913 (BMI)	0.903 (WEIGHT)	0.921 (WC)	4.144

*GWAS significance threshold ($p<5x10^{-8}$)

ALBU: albumin, ALKPHS: alkaline phosphatase, ALT: Alanine Aminotransferase, BF: body fat, BMI: body mass index, CHLOR: chloride, CHOL: total cholesterol, CHOLHDL: cholesterol HDL-C ratio, CREAT: serum creatinine, DBP: diastolic blood pressure, GGT: Gamma-glutamyl Transferase, GLOB: globulins, GLU: random glucose, HDL: HDL-cholesterol, HEIGHT: height, HIP: hip circumference, LDL: LDL-cholesterol, POTA: potassium, SBP: systolic blood pressure, SODIUM: sodium, TBILI: total bilirubin, TPROT: total protein, TRIG: triglyceride, UREA: serum urea ,WC: waist circumference, WEIGHT: weight, WHR: waist hip ratio

NA: not available

Table S6. Comparison between the inclusion and the removal of serum creatinine from PCAs.

Chr.	SNP	A1	A2	Original results with the inclusion of creatinine			Results after excluding creatinine		
				PC	Beta	P-value	PC	Beta	P-value
4	rs12640604	A	G	CGU-PC1	-0.344	$1.67x10^{-7}$	GU-PC1	-0.333	$9.48x10^{-8}$
				CGU-BH-PC1	-0.347	$3.37x10^{-8}$	GU-BH-PC2	-0.327	$3.56x10^{-8}$
				CGU-CHTW-PC2	-0.448	$1.59x10^{-9}$	GU-CHTW-PC2	-0.407	$7.67x10^{-10}$

Table S7. Summarized information of 7 populations in the UK biobank data used in the replication analysis.

Ethnic groups	Age - mean	Age - SD	n	Female	Male
African	51.0	7.9	2969	1439	1530
British	56.9	8.0	408329	220802	187527
Caribbean	52.7	8.1	4026	2536	1490
Chinese	53.9	8.3	27386	15901	11485
Indian	54.1	8.3	5398	2627	2771
Irish	56.2	8.1	12099	6276	5823
Pakistani	51.3	8.4	1643	639	1004

a.

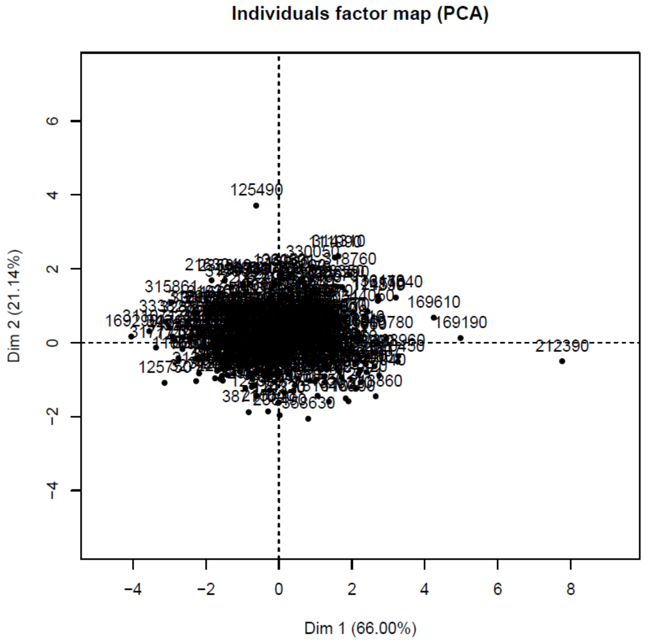

b.

Top 10 samples with the lowest eGFR

UUID	CREAT	eGFR	UREA
212390	188	34.8	10.9
169190	130	36.5	9.7
169610	110	36.7	9.9
312481	100	48.1	6.8
255670	89	48.5	6
301001	100	49.1	6.3
110390	100	49.8	7.1
212771	105	50.8	5.7
111890	90	52.0	9.3
320450	120	52.8	7.5

Figure S1. CGU-PC1 captured the most information on renal function. a) Individual factor map under CGU-PC1 (Dim 1) and CGU-PC2 (Dim 2). b) Top ten samples with the lowest eGFR measurements.

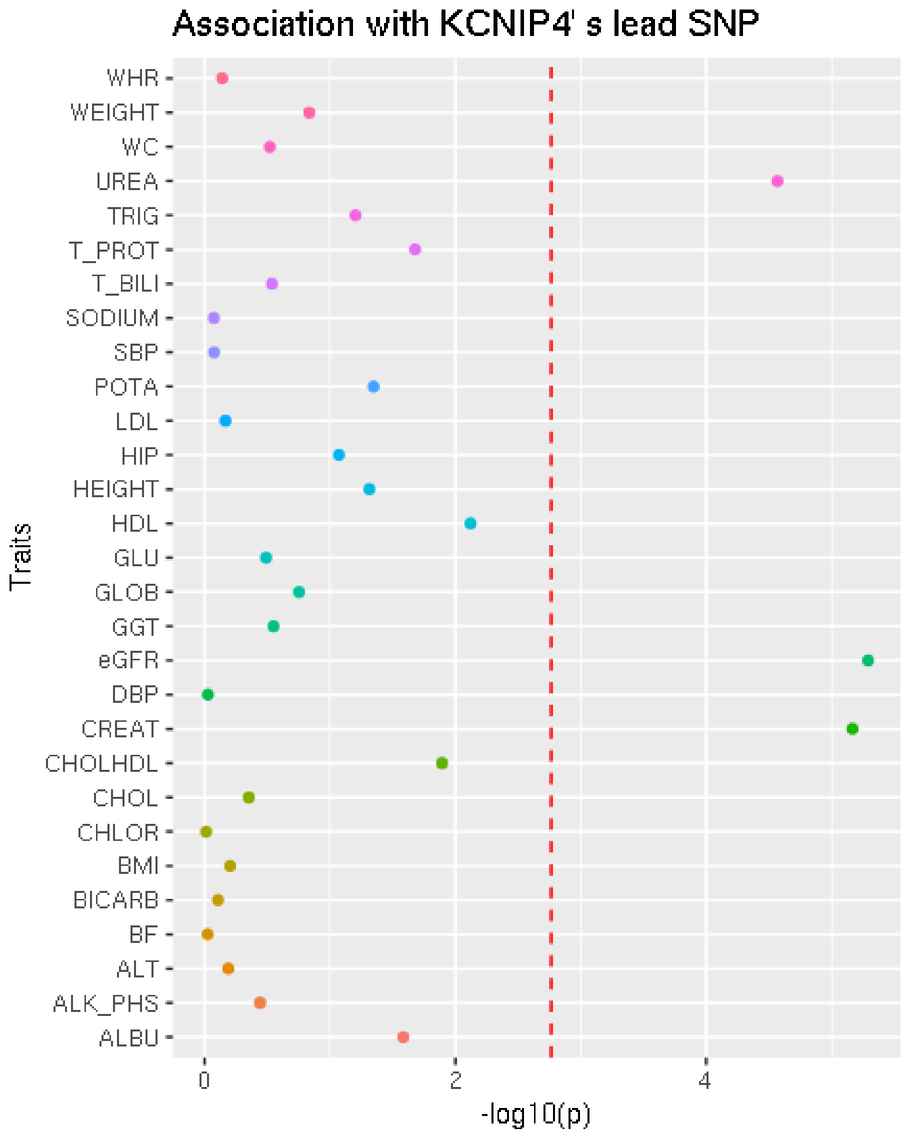

Figure S2. Association analysis of the lead SNP rs12640604 at *KCNIP4* locus with all the 29 phenotypic traits. The red line indicates the corrected p-value threshold of $1.7x10^{-3}$. Mixed linear model was applied in the association tests with age and sex as covariates and a GRM of whole genome information but without the lead SNP as random effect

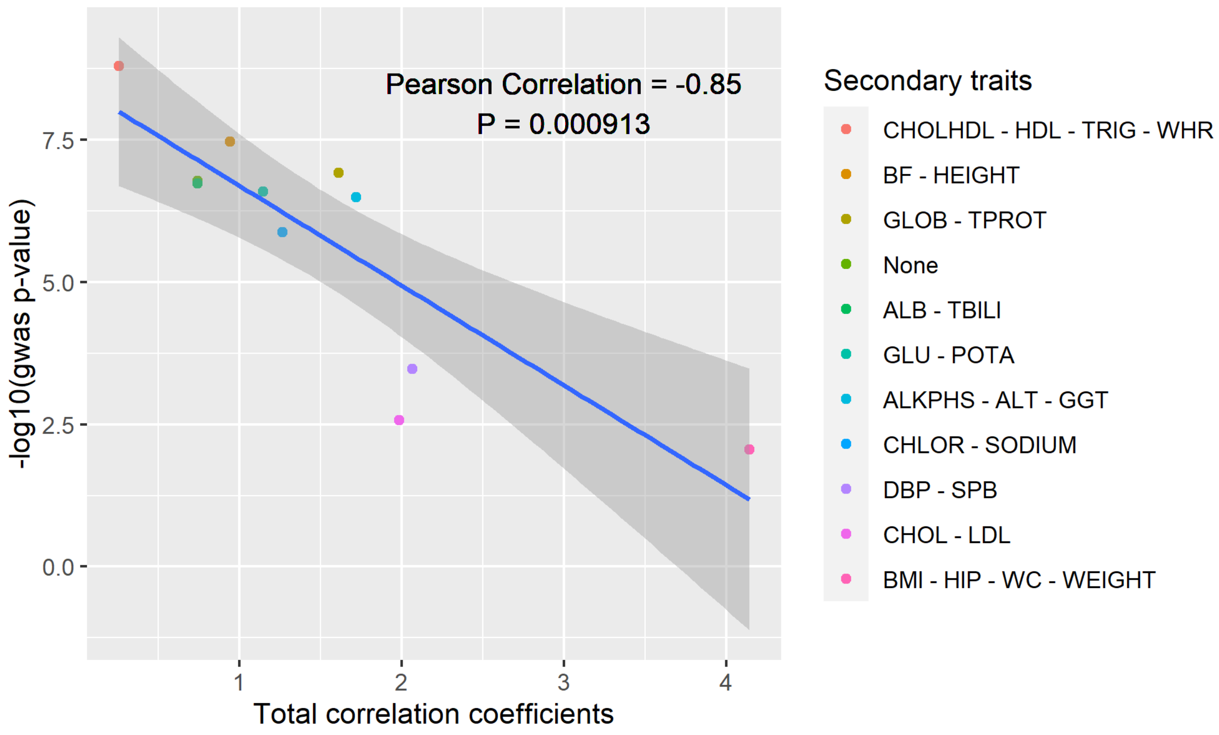

Figure S3. Correlation between GWAS p-values and the sums of correlation coefficients of component variables in each PC. The minimum p-values of the *KCNIP4* lead SNP rs12640604 were included from GWASs of each combination PCs and CGU-PCs. Refer to table S1 for the list of trait abbreviations.

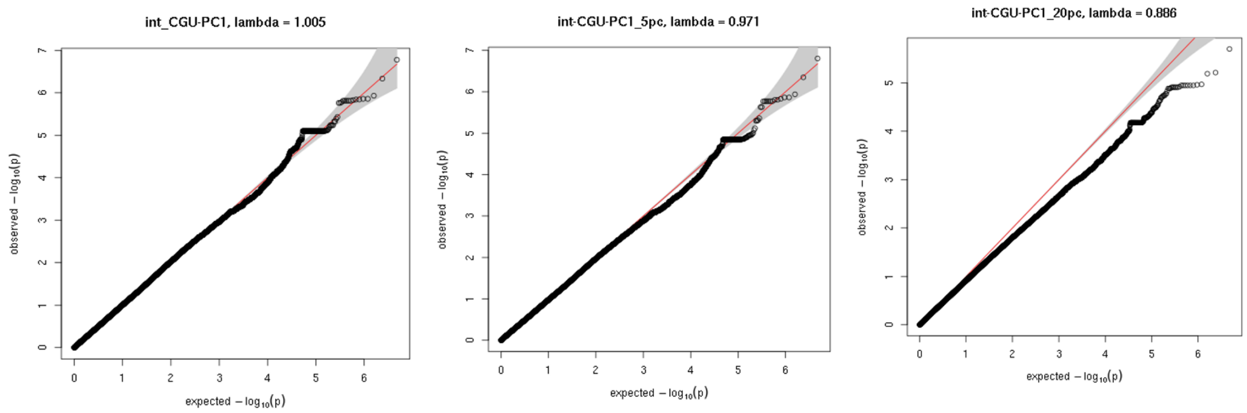

Figure S4. Quantile-quantile plots when 0, 5, and 20 principal components of genetic relationship matrix were included as covariates in GWAS of CGU-PC1.
